# Caloric restriction leading to attenuation of experimental Alzheimer's disease results from alterations in gut microbiome

**DOI:** 10.1111/cns.14823

**Published:** 2024-07-11

**Authors:** Junyu Chen, Cong Zou, Hongbing Guan, Xiaoming Zhou, Le Hou, Yayong Cui, Junhua Xu, Ping Luan, Dong Zheng

**Affiliations:** ^1^ Department of Neurology, The Affiliated Brain Hospital Guangzhou Medical University Guangzhou China; ^2^ Guangdong Yunzhao Medical Technology Co., Ltd. Guangzhou China; ^3^ School of Basic Medical Sciences Shenzhen University Shenzhen China

**Keywords:** Alzheimer's disease, amyloid β, caloric restriction, gut microbiome, intestinal flora, neuroinflammation

## Abstract

**Background:**

Caloric restriction (CR) might be effective for alleviating/preventing Alzheimer's disease (AD), but the biological mechanisms remain unclear. In the current study, we explored whether CR caused an alteration of gut microbiome and resulted in the attenuation of cognitive impairment of AD animal model.

**Methods:**

Thirty‐week‐old male APP/PS1 transgenic mice were used as AD models (AD mouse). CR was achieved by 30% reduction of daily free feeding (*ad libitum*, AL) amount. The mice were fed with CR protocol or AL protocol for six consecutive weeks.

**Results:**

We found that with CR treatment, AD mice showed improved ability of learning and spatial memory, and lower levels of Aβ40, Aβ42, IL‐1β, TNF‐α, and ROS in the brain. By sequencing 16S rDNA, we found that CR treatment resulted in significant diversity in composition and abundance of gut flora. At the phylum level, *Deferribacteres* (0.04%), *Patescibacteria* (0.14%), *Tenericutes* (0.03%), and *Verrucomicrobia* (0.5%) were significantly decreased in CR‐treated AD mice; at the genus level, *Dubosiella* (10.04%), *Faecalibaculum* (0.04%), and *Coriobacteriaceae UCG‐002* (0.01%) were significantly increased in CR‐treated AD mice by comparing with AL diet.

**Conclusions:**

Our results demonstrate that the attenuation of AD following CR treatment in APP/PS1 mice may result from alterations in the gut microbiome. Thus, gut flora could be a new target for AD prevention and therapy.

## Graphical abstract

In the experimental model of Alzheimer's disease, caloric restriction attenuated severity of the disease, improved ability of learning and spatial memory, and decreased Aβ expression. The caloric restriction resulted in significant alteration of composition and abundance of gut flora.

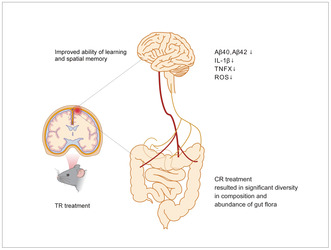

## 1 INTRODUCTION

Alzheimer's disease (AD) is a serious neurodegenerative disease whose main clinical manifestation is cognitive decline, accompanied by significant psychiatric and behavioral abnormalities.^1^, ^2^ AD is pathologically characterized by the accumulation of amyloid‐β peptide (Aβ) aggregates and neurofibrillary tangles that consist of hyperphosphorylated tau proteins.^3^ The amyloid‐beta (Aβ) cascade hypothesis is the most classic theory for the pathogenesis of Alzheimer's disease. However, these hypotheses cannot fully explain some phenomena in the occurrence and development of AD. The aggregation and activation of microglia and astrocytes can be observed around the plagues and tangles, the levels of the inflammatory cytokines and markers are increased,^4^, ^5^ and the identified risk genes of AD are found to be associated with the innate immune functions. All these evidences suggested that neuroinflammation may have an important role in the pathogenesis of AD.^6^ Moreover, accumulating evidences indicated that oxidative stress may be a key mechanism that causing cognitive aging and neurodegeneration and play a role in AD pathogenesis.^7^, ^8^ Accordingly, antiAβ, anti‐tau, and anti‐inflammatory strategies have been developed in clinical trials. Nevertheless, the non‐pharmacological therapeutic strategies for preventing the neurons from pathological death are still needed.

Diet can influence the neurophysiology. CR, characterized by the reduction of daily food (calories) intake by 20%–50%, is one of the most studied non‐pharmacological therapeutic interventions and is considered the most effective method of extending lifespan in mammals.^9^, ^10^ For example, CR of 30% reduction is found to extend lifespan to 10% level.^11^ The promising effects of CR in diabetes and cardiometabolic diseases are reported.^12^ With respect to the aging‐related neurodegenerative diseases, such as AD and Parkinson's disease, CR has also shown a role of delaying or preventing the development of the diseases.^13^, ^14^, ^15^ CR treatment in SD rats significantly attenuated acrolein‐induced cognitive impairment.^16^ On the other hand, a recent study in triple transgenic AD model mice showed that the high‐fat diet (with 60% fat) resulted in cognitive impairment accompanied with increased oxidative stress and neuronal cell death.^17^

The underlying mechanisms of the CR functioning on neurodegenerative disease are not clear so far. The reports showed that CR could suppress inflammatory responses and oxidative stress injury as well as improve cognitive impairment.^18^, ^19^ The burden of amyloid aggregates and tau protein was reduced while autophagy was increased along with CR treatment.^20^ Recently, a number of studies focused on the modulation of Nrf2, a transcription factor which plays a neuroprotection by suppressing oxidative stress damage. It was reported that CR activated Nrf2 and its signal pathway thereby eliciting protective effects.^21^

The importance of the gut–brain axis and gut microbiota in maintaining homeostasis of brain health has been appreciated. Gut microbial dysbiosis leads to the secretion of amyloid and lipopolysaccharides (LPS), which disturb the gastrointestinal permeability and blood–brain barrier thus influence the neuroinflammation and neuronal injury and cause neuronal death in AD. Recently, gut microbiome and probiotic intervention in AD has drawn much more attention.^22^ It was showed that CR could delay the development of AD and might be a result from alterations in the composition and metabolic function of the gut microbiome.^23^

Such reports suggest that CR could be a valuable strategy in the adjuvant therapy of central nervous diseases. However further study is needed for clarifying the exact mechanisms of the neuroprotective effect of CR treatment on AD. In the current report, we tried to understand the interaction between CR, gut microbiota, and the inflammatory signaling pathway in an AD mouse model. The results may provide evidences in developing a more effective protocol for CR therapy for AD.

## 2 MATERIALS AND METHODS

### 2.1 Animal and caloric restriction intervention

Male 30‐week‐old APP/PS1 mice (C57BL/6 background) and age‐ and gender‐matched C57BL/6 littermates (WT) were purchased from Carvensberg Model Animal Research Co. Ltd (Suzhou, China). Mouse food was purchased from Synergy biological. Mice were housed individually in a SPF facility. The experiments were approved by the Animal Ethics Committee of Guangzhou Medical University.

The APP/PS1 and WT mice were fed with two protocols: AL or CR. All mice were fed AL 1 week before applying CR protocol, the average amount of food taken by each mouse was calculated daily; for CR intervention, the mice were fed with 70% amount of the daily AL amount. The CR treatment were maintained for six consecutive weeks while the control groups kept AL feeding protocol. The weights of the mice were measured and recorded daily.

### 2.2 Water maze experiment

The experiment consisted of two sessions of experiments: position navigation training and space exploration. Before the experiment, the mice were first allowed to acclimate to the platform for 20 sec, then they were placed in the circular pool from the entry point of each quadrant. An automatic end time of 120 sec was set for position navigation training. If the mouse successfully found the platform and stayed there for more than 2 sec, the stay time was recorded. If the mice failed to find the platform after 120 sec, the experimenter would guide them to the platform and leave them there for 15 sec to familiarize with the environment before removing the mice. At the end of each trial, the mice were dried off with a clean towel. Each mouse was tested four times a day (once in each of the four quadrants), with 15 min intervals for five consecutive days. The space exploration experiment was then conducted on day 6, with the other environmental factors unchanged except that the platform was removed. Mice in each group were placed in the water in the opposite quadrant to the target quadrant (i.e., the quadrant where the platform was located), and the end time was set to 60 sec. The mice were allowed to swim freely during the set time, and explore the location of the platform based on the spatial memory they had acquired during the previous 5 days of training.

### 2.3 Enzyme‐linked immunosorbent assay (ELISA)

The hippocampi were collected and stored at −80°C. The frozen hippocampi were homogenized in RIPA buffer, sonicated and centrifuged. The supernatant was collected and measured for Aβ40, Aβ42, IL‐1β, ROS, and TNF‐α by ELISA using ELISA kit (Sinobestbio, China) according to the manufacturer's instructions.

### 2.4 Fecal 16S rDNA sequencing techniques and methods

On the last day of the sixth week of intervention, fecal samples of the four groups of mice were collected at the same time, and the fecal samples of each mouse were sequenced uniformly. All the sequencing results were simulated and analyzed by QIIME2 software. Follow the instructions of the E.Z.N.A.®Stool DNA Kit to extract fecal genomic DNA step by step. DNA was quantified by 1% agarose gel electrophoresis. The V3‐V4 variable region of 16S rDNA was selected as the PCR amplification region of bacterial genomic DNA. The sequence was amplified by PCR using universal primers 341F (5′‐CCTACGGGNGGCWGCAG‐3′) and 805R (5’‐ACTACHVGGGTATCTAATCC‐3′), and the PCR products were confirmed by electrophoresis. Subsequently, the PCR products were purified by AMPure XT beads (Beckman Coulter Genomics, Danvers, MA, USA) and quantified by Qubit (Invitrogen, USA). Amplicon pools were used for sequencing, and the size and number of amplicon libraries were evaluated on Agilent 2100 Bioanalyzer (Agilent, USA) and Illumina's library quantification kit (Kapa Biosciences, Woburn, MA, USA). The library is sorted on the NovaSeq PE250 platform. Then, Vsearch software was used to filter and optimize chimeric sequences. DADA2 is used to demodulate the sequence and get the feature list and feature sequence. Then, the operational taxonomic units (OTU) table is constructed to obtain the final feature table and feature sequence for further analysis. Finally, according to SILVA (release132) classifier, the relative abundance of each sample was used to normalize the feature abundance. The analysis of microbial diversity mainly focuses on alpha (α) diversity and beta (β) diversity. α Diversity is mainly used to reflect species richness, evenness, and sequencing depth, including chao1, observed species, goods coverage, Shannon, and Simpson. β Diversity reflects species differences among different samples, which are mainly reflected by principal component analysis (PCA), principal coordinate analysis (PCoA), similarity analysis (ANOSIM), non‐multidimensional scaling analysis (NMDS), cluster analysis (UPGMA), and multivariate analysis of variance (PermANOVA).

### 2.5 Statistical analysis

Data analysis was performed using SPSS 20.0 software. The latency in position navigation training was analyzed using repeated measures analysis of variance (rm‐ANOVA). The space exploration data were analyzed using one‐way analysis of variance (one‐way ANOVA), followed by pairwise least significant difference (LSD) comparisons. The other data were analyzed using Student's *t*‐test. The results were expressed as mean ± standard deviation (SD). The *p* value < 0.05 was considered statistically significant.

## 3 RESULTS

### 3.1 CR did not cause the malnutrition

**FIGURE 1:**
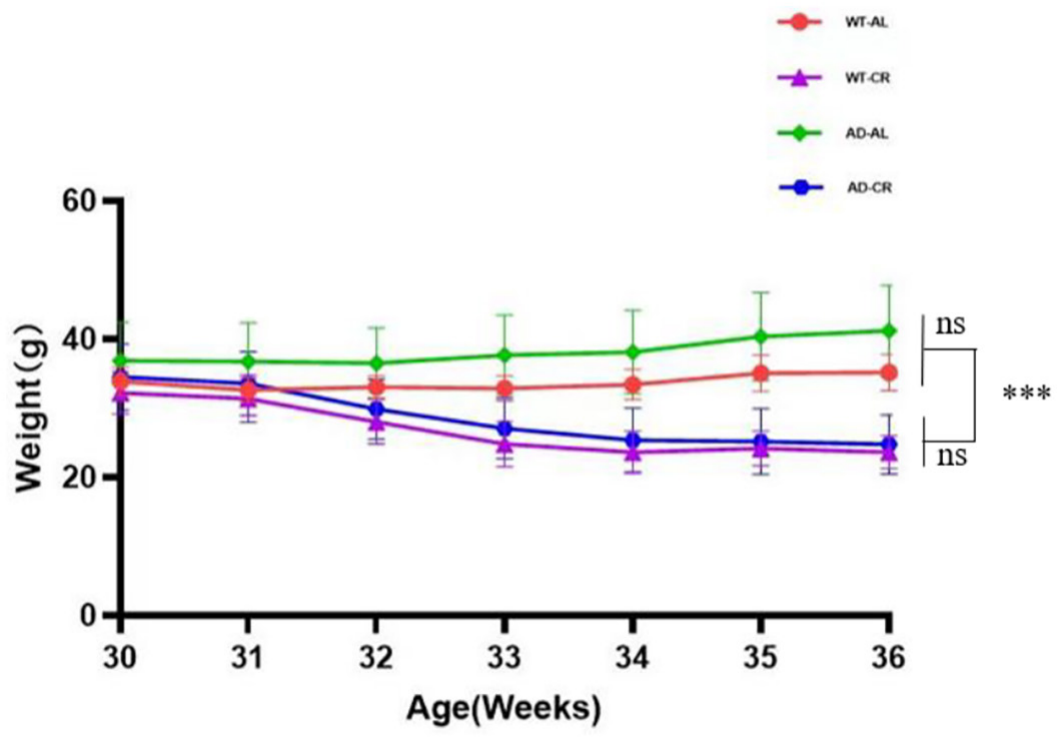
CR protocol did not cause malnutrition. The mice were fed according to the different protocols. The weight was measured at the end of each week after treatment. The weights in each group were expressed as Mean ± SD with five to seven mice per group. There were significant differences between the AL and CR groups, ***p < 0.01. There were no significant differences between WT‐AL and AD‐AL; also, there were no significant differences between WT‐CR and AD‐CR, ns: *p* > 0.05.

Mice were fed with the different protocols as described in Materials and Methods and divided into the different groups accordingly: WT‐AL, wild‐type mice with AL protocol; WT‐CR, wild‐type mice with CR protocol; AD‐AL, AD model with AL protocol; AD‐CR, AD model with CR protocol. We started the CR protocol at the first day of 30 weeks age, and continued six consecutive weeks. To evaluate whether CR would lead to malnutrition, we monitored the weight changes of each mouse. The weight of each mouse was measured at the last day of each week. The average weight in each group was expressed as shown in Figure 1. We found that the average weight in the AD‐CR and WT‐CR groups decreased started from 32nd week, but remained stable from 34th to 36th week. There were no significant differences between WT‐AL with WT‐CR n or AD‐AL with AD‐CR. The results indicated that CR treatment did not cause the malnutrition.

### 3.2 CR improved learning and memory

**FIGURE 2:**
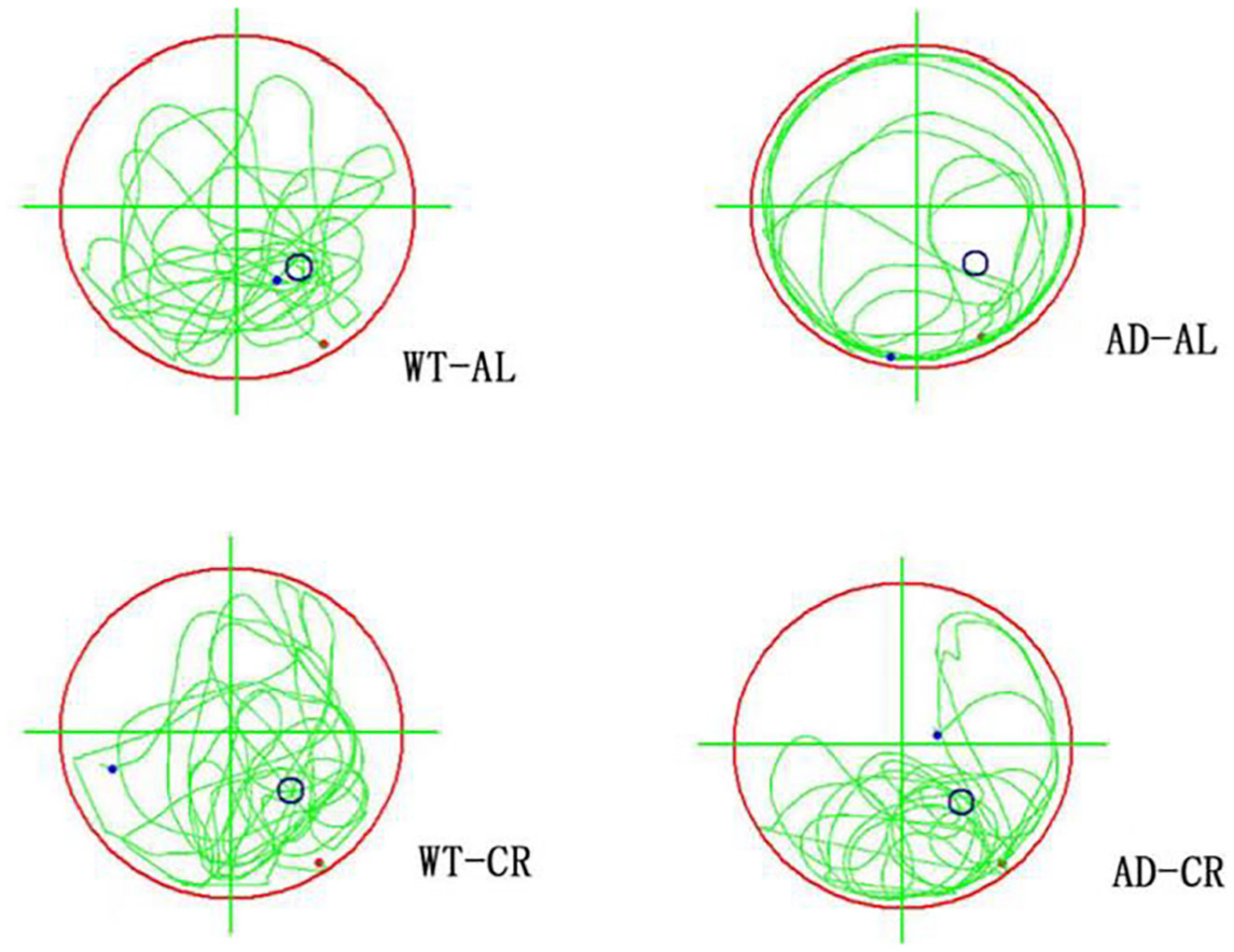
Representative path‐tracking patterns for each group of mice in the spatial exploration experiment. The green curve represents the path of mice in the probing trial to the platform (black circle), the histogram shows the repeated numbers across the platform of the mice. Five to seven experimental animals per group. The form of movement of the mouse in each group on the sixth day.

We performed the water maze experiments to test learning and memory function. In the navigation training experiment, we recorded the path tracking patterns of mice in each quadrant of the swimming pool during the probing trial to the platform. In comparison with the WT groups (WT‐AL and WT‐CR), the AD‐AL mice were likely to probe around the pool wall, which was consistent with the swimming characteristics of the AD model. The AD‐AL group crossed the platform less frequently compared to WT‐AL and WT‐CR whereas AD‐CR mice crossed the platform more frequently than the AD‐AL group to the same level as the WT‐AL and WT‐CR groups, which demonstrated that CR treatment recovered the memory of AD mice. To compare the probing patterns, the two protocols did not cause the abnormal tracking patterns in the WT‐AL and WT‐CR groups whereas the AD‐AL group showed an abnormal pattern. Again, CR treatment recovered the normal patterns as same as the WT groups (Figure 2).

**FIGURE 3:**
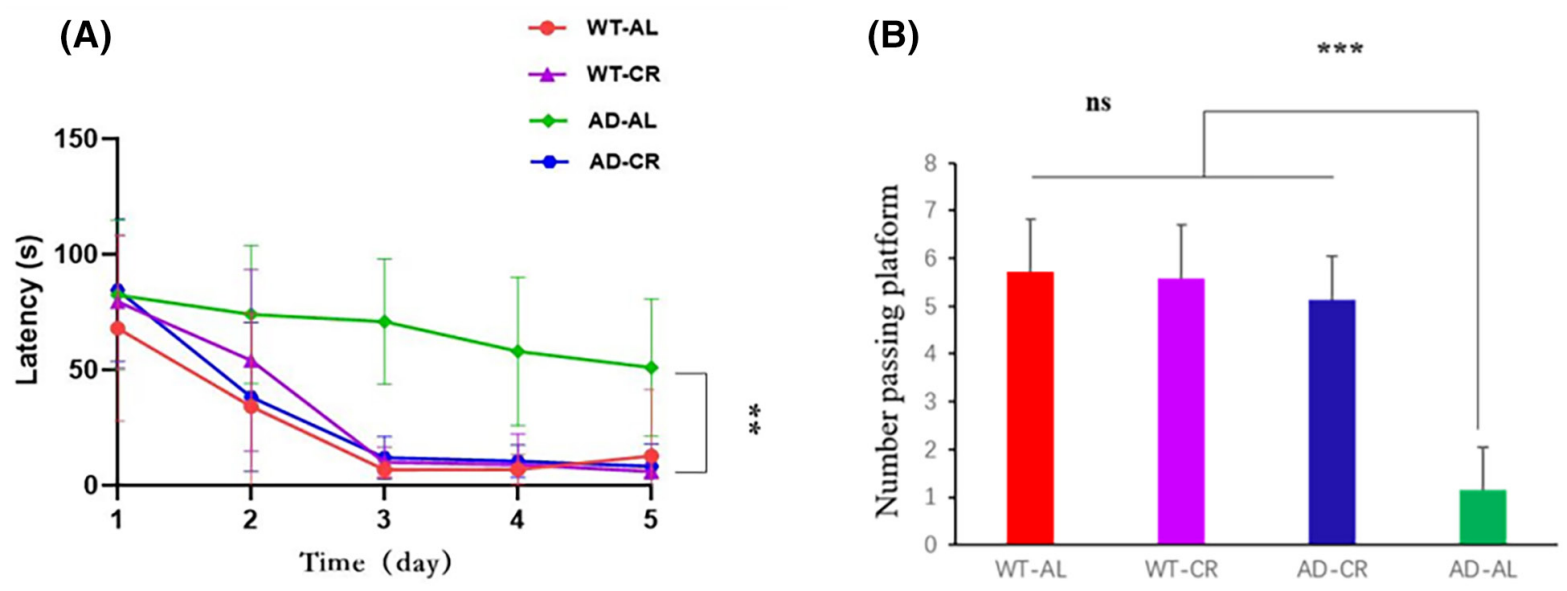
CR protocol significantly recovered the latency of AD mice. In the position navigation training experiments, the latency (sec) was recorded in the different groups. The results were expressed as mean ± SD with five to seven mice per group. **p < 0.05, ***p < 0.01. ns, no significant difference; *p* > 0.05. There were no significant differences among groups of WT‐AL, WT‐CR, and AD‐CR (*p* > 0.05). On the other hand, there was a significant difference between AD‐AL with the other three groups (*p* < 0.05). (A) Five‐day latency (the average of each group). (B) The number of times crossing the plate. The number of times each group crossed the plate on the fifth day.

The time to start the tracking path (latency period) is the important factor to judge the learning and memory capability. As shown in Figure 3, the two feeding protocols maintained the normal latency period in the WT groups (WT‐AL and WT‐CR). However, AL protocol caused the significantly delayed latency period in AD mice (AD‐AL). Furthermore, CR protocol in the AD mice (AD‐CR) significantly reversed the delayed latency period to the normal level as same as the WT groups (WT‐AL and WT‐CR). From the second day, the latency period of the mice in the AD‐AL group was significantly delayed comparing to the other groups, indicating AD mice successfully exhibited the characteristics of AD and were suitable models for CR application (Figure 3A). This result was also reflected in the number of repeated passing the platform (Figure 3B).

**FIGURE 4:**
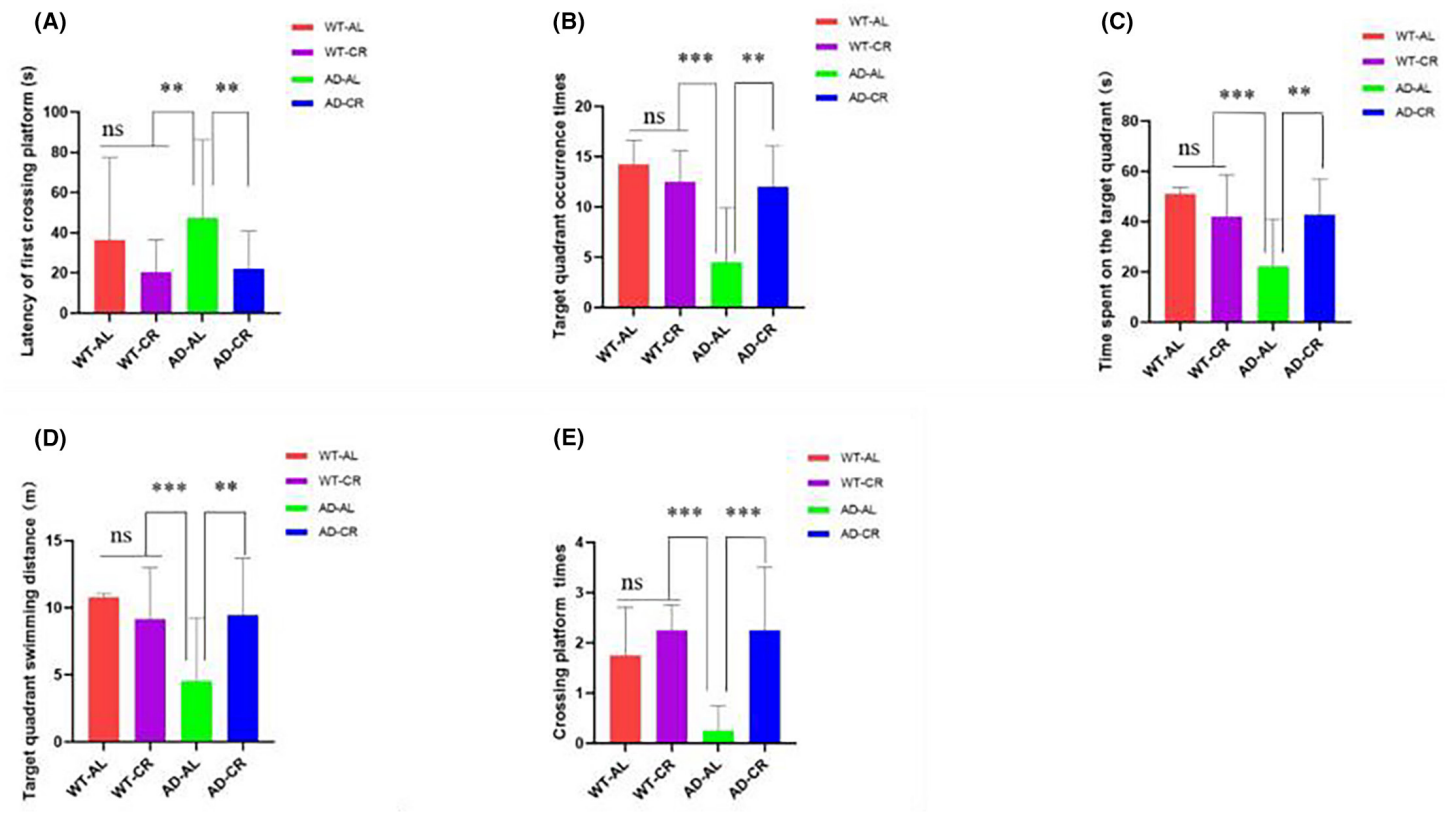
Main assessment indicators during space exploration by mice in each group. (A) Latency to first platform crossing; (B) The number of times appearing in the target quadrant; (C) Time spent in the target quadrant; (D) Swimming distance in the target quadrant; (E) Number of platform crossings. **p < 0.05, ***p < 0.01. ns, no significant difference; *p* > 0.05. Five to seven experimental animals per group. On the sixth day, the motion parameters of mice in each group.

The main factors of water maze experiments such as latency period, occurrence and spent time on the target quadrants, swimming distance in the quadrants, and numbers crossing the platform are shown in Table 1 and analyzed in Figure 4. For the latency, there were no significant differences among the WT‐AL, WT‐CR, and AD‐CR groups whereas the AD‐AL group showed the significant increase of the latency (Figure 4A). Meanwhile, the number of occurrences in the target quadrant, the time spent in the target quadrant, the swimming distance in the target quadrant, and the number of crossing the platform were significantly lower in the AD‐AL group than the other groups. (Figure 4B–E).

**TABLE 1:** Main evaluation indicators of each group of mice in the stage of spatial exploration.

Main index grouping	Number of appearing in the target quadrant	Target quadrant swimming distance (m)	Target quadrant time (s)	Number of platform crossings	Latency to first platform crossing (s)
WT‐AL	14.3 ± 2.36	10.8 ± 0.28	51.1 ± 2.53	1.8 ± 0.96	36.4 ± 4.05
WT‐CR	12.5 ± 3.11	8.5 ± 4.11	42.1 ± 6.46	2.3 ± 0.5	20.5 ± 5.94
AD‐AL	4.5 ± 0.45*	4.5 ± 0.7*	22.2 ± 8.72*	0.3 ± 0.5*	47.3 ± 8.96*
AD‐CR	12 ± 4.98**	9.5 ± 2.22	42.7 ± 4.21	2.3 ± 1.26**	22.1 ± 8.82

*Note*: Results are expressed as mean ± SD with five to seven mice per group.

* *p* < 0.05.

** *p* < 0.05.

### 3.3 CR reduced Aβ expression in hippocampi of AD mice

**FIGURE 5:**
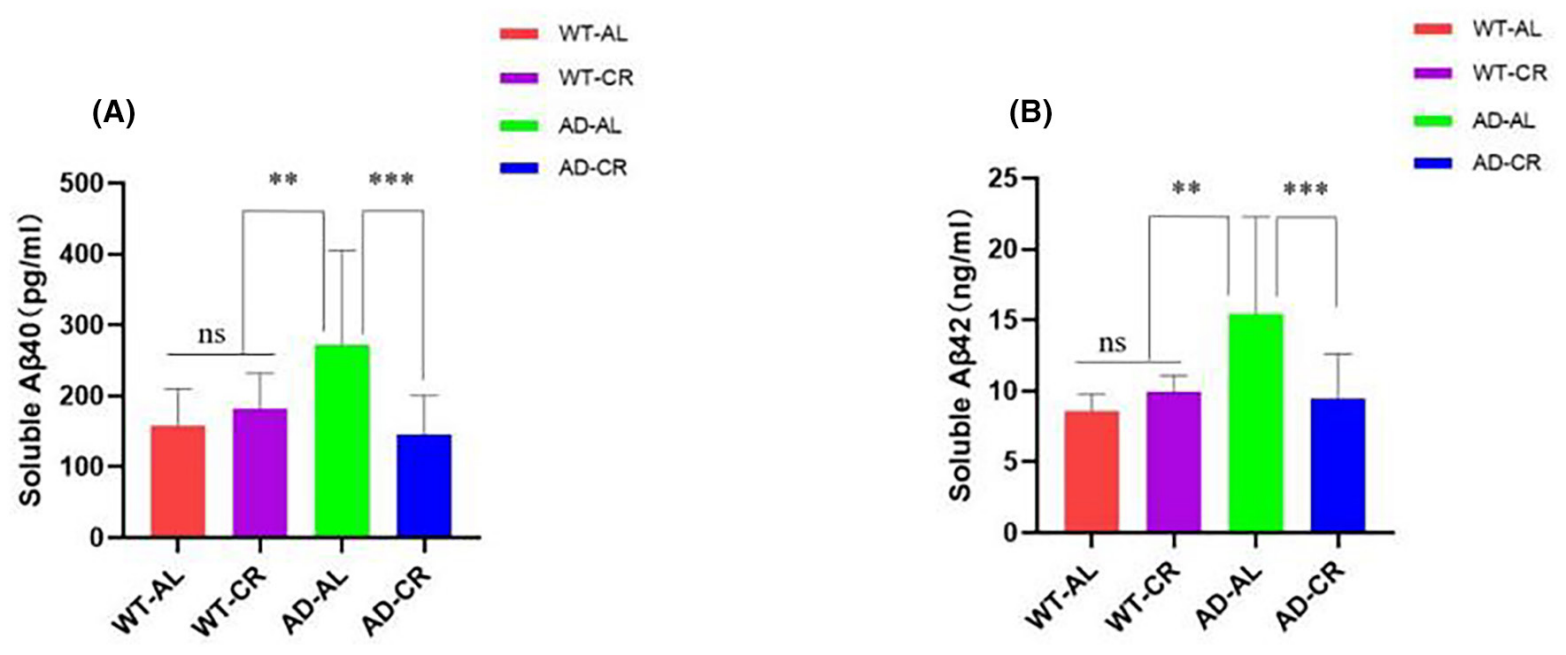
Soluble (A) Aβ40 and (B) Aβ42 levels in the hippocampus. **p<0.05, ***p<0.01. ns, no significant difference; *p* > 0.05. The results were expressed as mean ± SD with five to seven mice per group.

In the AD mouse model, Aβ 40 and Aβ 42 expression can be detected as early as 12 weeks, and a large amount of Aβ aggregates are found in the brains at 24–32 weeks, their numbers are increased with age.^24^, ^25^, ^26^ To find where CR could affect the expression of Aβ, we measured Aβ levels in the hippocampi by ELISA. The results showed that the levels of Aβ40 and Aβ42 were significantly higher in the AD‐AL group than those in the other groups. CR treatment reduced Aβ expression; no significant differences were found between the AD‐CR and WT groups (Figure 5A,B).

### 3.4 CR suppressed the expression of the pro‐inflammatory factors and alleviated neuroinflammation in AD mice

**FIGURE 6:**
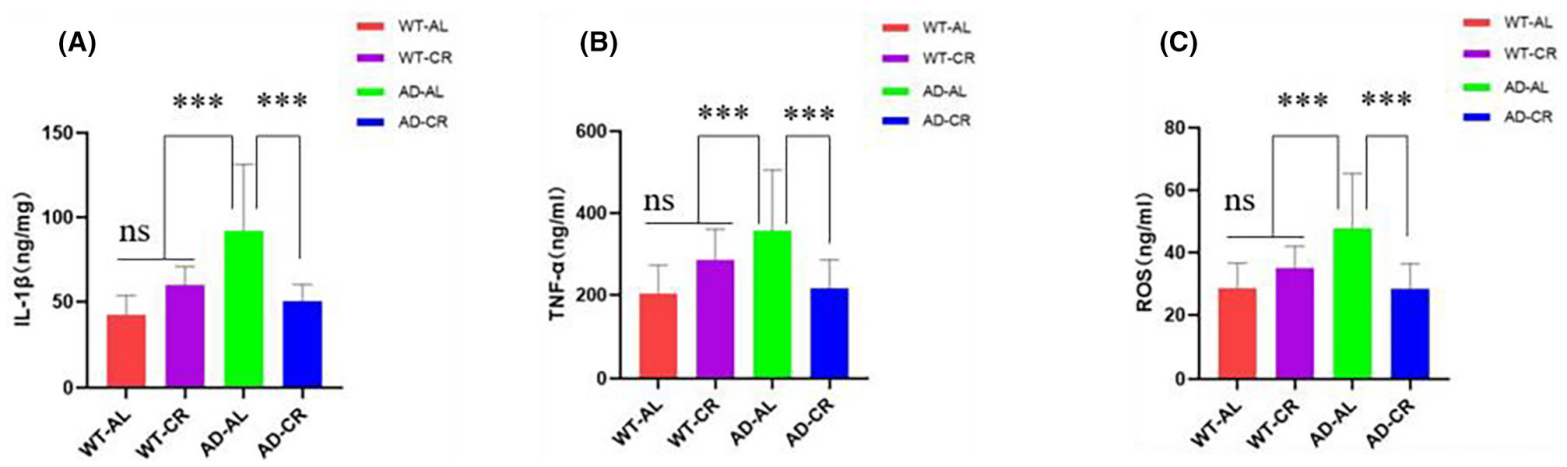
Levels of pro‐inflammatory factors and ROS. Expression levels of (A) IL‐1β, (B) TNF‐α, and levels of (C) ROS in the hippocampus were detected by ELISA. Briefly, hippocampus brain tissue was made into tissue homogenate, and then samples were used for detection of reactive oxygen species following instructions of the ROS kit. In the AD‐CR group, the expression of IL‐1β, TNF‐α, and ROS was significantly suppressed indicated by the reduced levels. The results were expressed as mean ± SD with five to seven mice per group. ***p<0.01. ns, no significant difference; *p* > 0.05.

To examine whether CR could effectively suppress the expression of the proinflammatory factors in the brains of AD mice, we examined the levels of pro‐inflammatory factors and reactive oxygen species (ROS) in the hippocampi by ELISA. The results showed that the levels of IL‐1β, TNF‐α, and ROS were much higher in the AD‐AL group whereas CR treatment suppressed the expressions as the levels were much lower in the AD‐CR group (Figure 6 **)**. The results demonstrated that CR could inhibit the progression of neuroinflammation.

### 3.5 CR altered the composition of gut microbiota in mice

**FIGURE 7:**
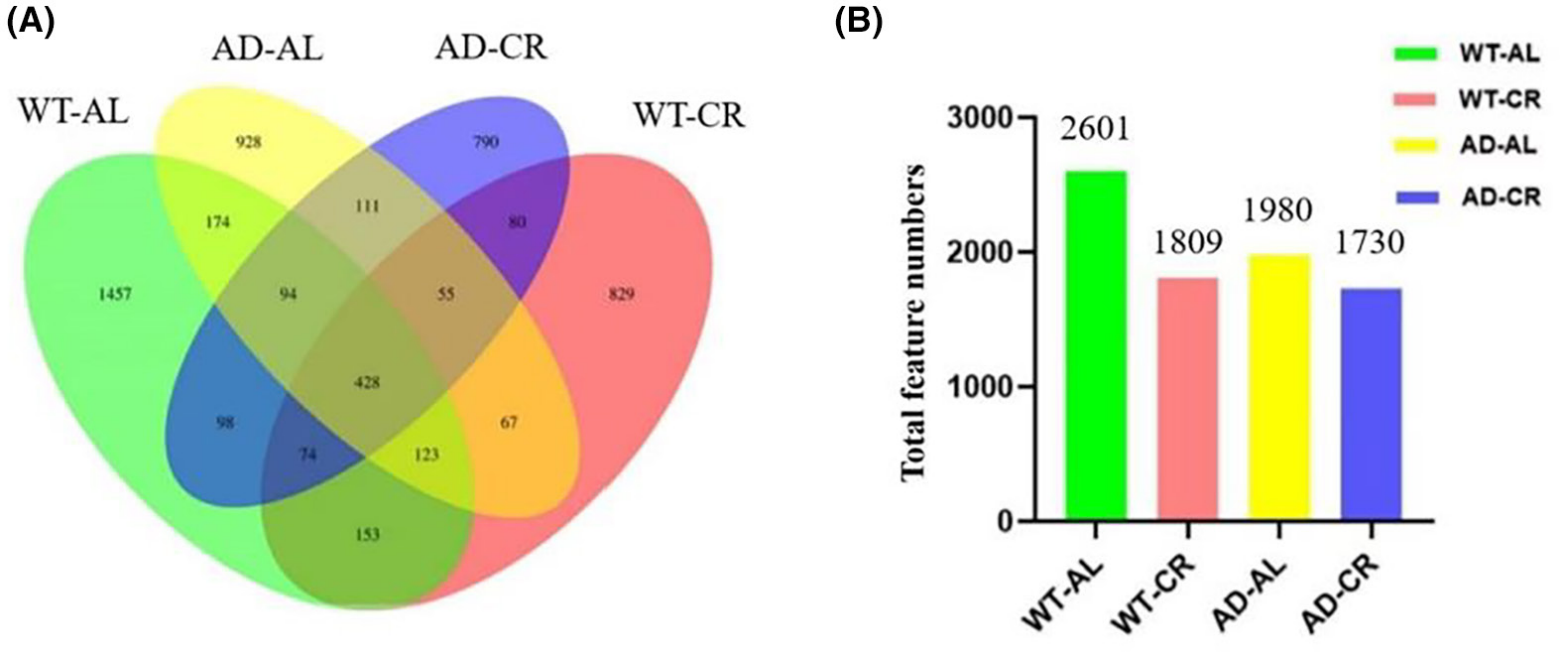
Effects of CR on intestinal microflora feature complexity in AD mice. According to Venn diagram, there are some similarities and differences among the groups. (A) Venn diagram can visually present the number of common and unique features of fecal samples in each group at the OTU‐like level. Ovals with different colors represent different experimental groups, and the numbers are the numbers of common or unique features between groups. (B) Feature diversity shows CR intervention changed the feature of intestinal flora in mice. The total numbers in each group were also labeled in the corresponding column.

CR changed the types of intestinal flora in mice. Species Venn diagram analysis can visually present the number of common and unique features of fecal samples in each group at the OTU‐like level. As shown in Figure 7A, Ovals with different colors represent samples from different experimental groups, and the number is the number of common or unique features between groups. In Figure 7B, bar graphs with different colors represent total feature numbers from different experimental groups. According to Venn diagram, there are some similarities and differences among the groups. A total of 5461 features were obtained from the four groups of samples. Among them, there are 428 features shared by the four groups of samples, accounting for about 7.8%. There were 2943 different feature numbers between the AD‐Al group and the WT‐AL group, indicating that the intestinal flora of the AD model mice was different from that of wild‐type mice. The number of non‐common features in the WT‐CR and WT‐AL groups was 2854. The number of non‐common features in the AD‐CR and AD‐AL groups was 2334, indicating that CR changed the types of intestinal flora in mice.

### 3.6 CR altered α diversity and β diversity of intestinal flora in mice

α Diversity includes chao1, observed species, Shannon, and Simpson. The first two mainly reflect the richness of microbial species in the samples, and the larger the value, the more species. The latter two mainly measure the diversity of species, and the larger the value, the higher the diversity of colonies. As shown in Table 2, we observed the CR intervention decreased levels of the Chao1 and Observed species while maintained the level of Shannon and Simpson. Our results suggested that CR protocol could affect the abundance of intestinal flora in AD mice.

**TABLE 2:** α Diversity index statistics. The values of chao1, observed species, Shannon, and Simpson are shown. In comparison with the AL protocol groups, the CR protocol groups showed significantly decreased values of chao1 and observed species (**p < 0.05), there was no significant difference in Shannon and Simpson values (p > 0.05).

	Chao1	Observed species	Shannon	Simpson
WT‐AL	626.9 ± 79.86	604 ± 76.98	6.9 ± 0.63	0.97 ± 0.022
WT‐CR	437.1 ± 39.29**	423.9 ± 33.0**	5.8 ± 0.54**	0.93 ± 0.036**
AD‐AL	528.3 ± 80.5	506.4 ± 75.1	6.5 ± 0.57	0.96 ± 0.019
AD‐CR	473.9 ± 83.54**	460.1 ± 78.74**	6.2 ± 0.63	0.95 ± 0.031

*Note*: Values are expressed as mean ± SD with five to seven mice per group.

**FIGURE 8:**
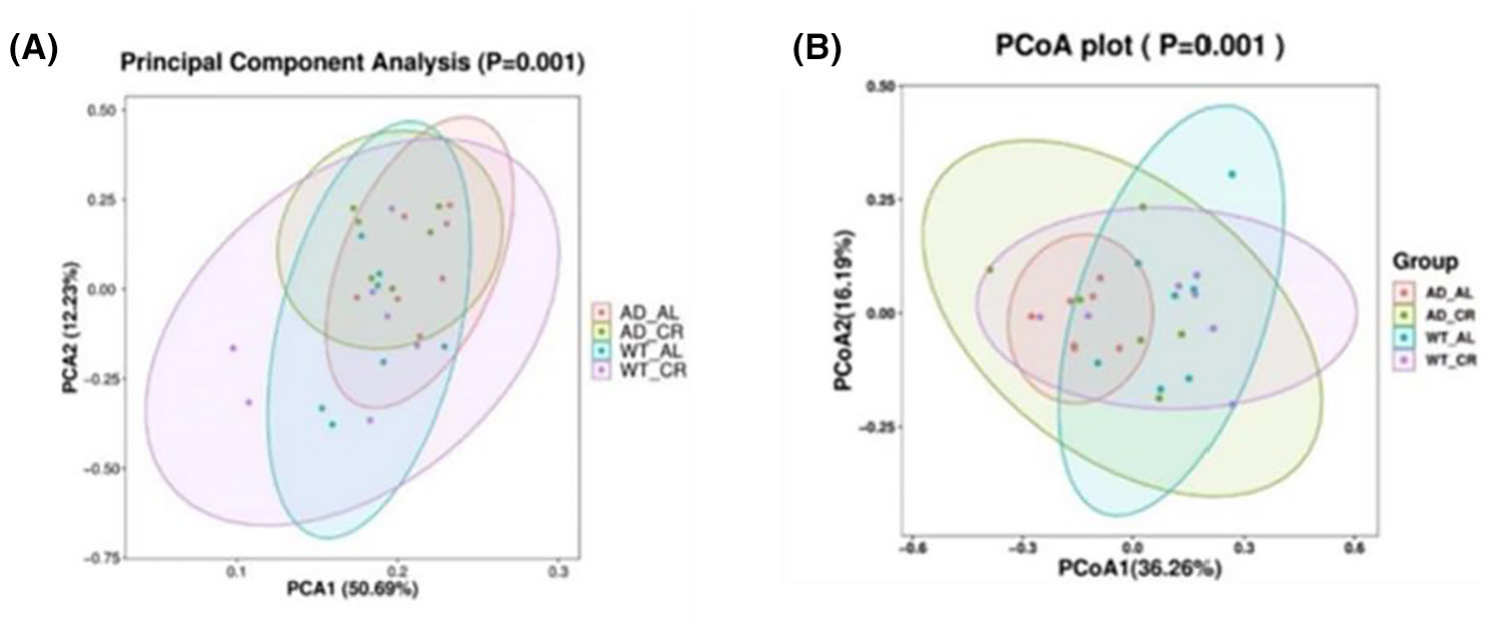
There were significant differences in the composition of microflora among the samples and in species diversity between groups. (A) PCA analysis: PCA1 (50.69%) and PCA2 (12.23%) were taken as the horizontal and vertical coordinates, the samples of each group were well separated, indicating that there were significant differences in the composition of microflora among the samples of each group. The dots represent different samples. (B) PCoA1 analysis: PCoA1(36.26%) and PCoA2(16.19%) were used as the horizontal and vertical coordinates, the overall distance of samples in the coordinate graph was far away, suggesting that there was a great difference in species diversity among groups. The dots represent different samples.

The β diversity analysis mainly reflected the species diversity among different samples. In this experiment, principal component analysis (PCA) and principal coordinate analysis (PCoA) were used for analyses. PCA and PCoA are methods those reduce the dimension of high‐dimensional feature space, and then reflect the difference of samples among groups on two‐dimensional coordinate graph by the algorithm. The closer the distance between the groups of samples in the coordinate diagram, the more similar the species composition of the samples. PCA analysis results of mice in each group in this experiment were shown in Figure 8A. When PCA1 (50.69%) and PCA2 (12.23%) were taken as horizontal and vertical coordinates, samples in each group were well separated, indicating obvious differences in the composition of microflora among samples in each group. As shown in Figure 8B, when PcoA1(36.26%) and PcoA2(16.19%) were taken as horizontal and vertical coordinates, the overall distance of samples in each group was far away from each other in the coordinate graph, suggesting that there was a large difference in species diversity among groups.

### 3.7 Diversity and taxonomic overview of the gut microbiome with CR intervention

**FIGURE 9:**
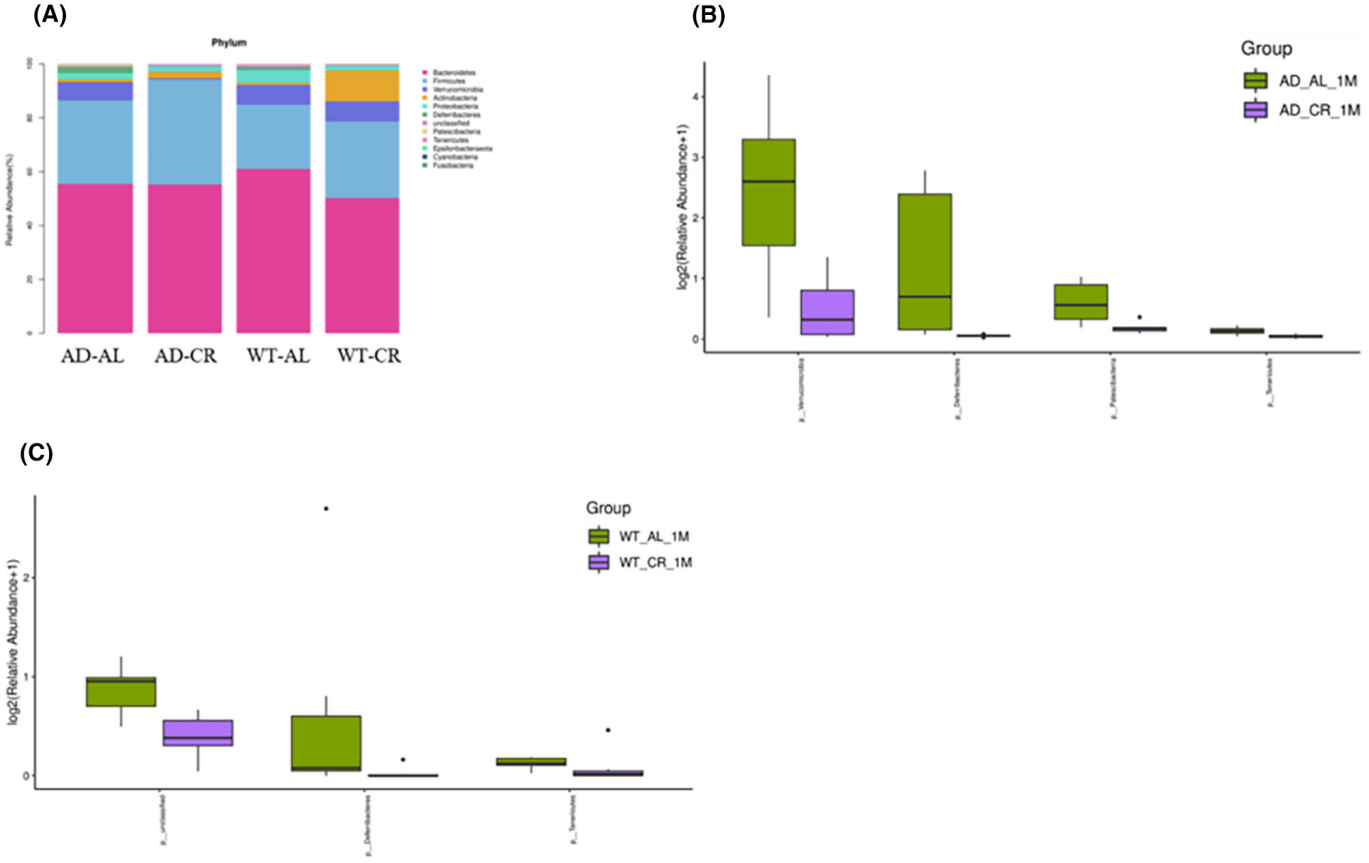
At the phylum level, the abundance of *Firmicutes*, *Verrucomicrobia*, and *Actinobacteria* changed significantly in the CR intervention group. *Bacteroidetes* and *Firmicutes* had the highest abundance in the intestinal flora of mice in all groups in this study. In addition, The relatively high abundance of bacteria were successively *Verrucomicrobia*, *Actinobacteria*, *Proteobacteria*, *Deferribacteres*, *Patescibacteria*, *Tenericutes*, *Epsilonbacteraeota*, *Cyanobacteria*, and *Fusobacteria*. Compared with the control group, the *Deferribacteres* (0.04%), *Patescibacteria* (0.14%), *Tenericutes* (0.03%), and *Verrucomicrobia* (0.5%) in the AD‐CR group were decreased (*p* < 0.05). *Deferribacteres* (0.02%) and *Tenericutes* (0.06%) in the WT‐CR group were decreased (*p* < 0.05).

At the phylum level, *Bacteroidetes*, *Firmicutes*, and *Verrucomicrobia* had the top three highest abundance in the intestinal flora of mice in all groups in this study. They were 55.45%, 31.07%, and 6.78% respectively in the AD‐AL group; 55.23%, 38.96%, and 0.5% respectively in the AD‐CR group; 61.01%, 23.86%, and 7.73% respectively in the WT‐AL group; 50.22%, 28.43%, and 7.50% respectively in the WT‐CR group. Successively, the relatively high abundance of bacterias were *Actinobacteria*, *Proteobacteria*, *Deferribacteres*, *Patescibacteria*, *Tenericutes*, *Epsilonbacteraeota*, *Cyanobacteria*, and *Fusobacteria* (Figure 9A). Compared with the AD‐AL group, *Deferribacteres* (0.04%), *Patescibacteria* (0.14%), *Tenericutes* (0.03%), and *Verrucomicrobia* (0.5%) were significantly decreased in the AD‐CR group (*p* < 0.05) (Figure 9B). Compared with the WT‐AL group, *Deferribacteres* (0.02%) and *Tenericutes* (0.06%) were significantly decreased in the WT‐CR group (*p* < 0.05) (Figure 9C).

**FIGURE 10:**
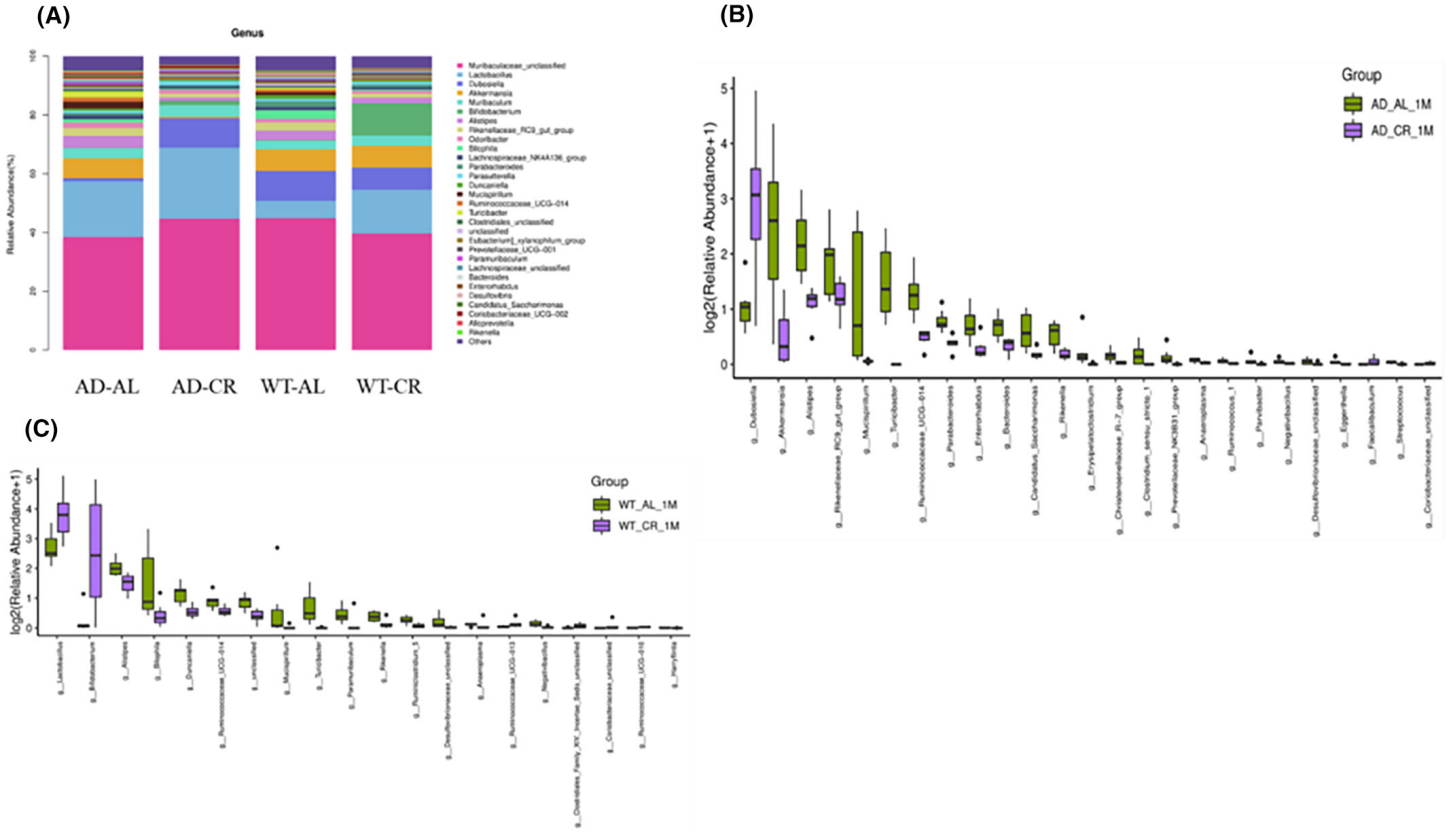
At the genus level, the abundance of *Lactobacillus*, *Dubosiella*, *Akkermansia*, and *Bifidobacterium* changed significantly in the CR intervention group. The top 10 taxonomic annotated species of intestinal microorganisms in mice of all groups in this experiment are *Muribaculaceae unclassified*, *Lactobacillus*, *Dubosiella*, *Akkermansia*, *Muribaculum*, *Bifidobacterium*, *Alistipes*, *Lianaceae RC9 intestinal group*, *Odoribacter*, *Bilophila*, etc. Compared to the control group, intestinal *Dubosiella* (10.04%) and *Coriobacteriaceae UCG‐002* (0.01%) were all increased in the AD‐CR group (*p* < 0.05). *Ruminococcaceae UCG014* (0.4%), *Alistipes* (1.16%), *Parabacteroides* (0.30%), and *Bacteroides* (0.27%) were all decreased in the AD‐CR group (*p* < 0.05). Compared with the control group, *Lactobacillus* (14.92%) and *bifidobacterium* (11.2%) in the WT‐CR group were increased (*p* < 0.05). *Ruminococcaceae UCG014* (0.49%) and *Lianaceae RC9 intestinal group* (1.88%) were lower in the WT‐CR group (*p* < 0.05).

At the genus level, the top 10 taxonomic annotated species of intestinal microorganisms in mice of all the groups in this experiment were *Muribaculaceae unclassified*, *Lactobacillus*, *Dubosiella*, *Akkermansia*, *Muribaculum*, *Bifidobacterium*, *Alistipes*, *Lianaceae RC9 intestinal group*, *Odoribacter*, and *Bilophila* (Figure 10A). Compared with the AD‐AL group, *Dubosiella* (10.04%), *Faecalibaculum*(0.04%), and *Coriobacteriaceae UCG‐002* (0.01%) were significantly increased in the AD‐CR group (*p* < 0.05). *Ruminococcaceae UCG014* (0.4%), *Alistipes* (1.16%), *Parabacteroides* (0.30%), and *Bacteroides* (0.27%) were all decreased in the AD‐CR group (*p* < 0.05) (Figure 10B). Compared with the WT‐AL group, *Lactobacillus* (14.92%) and *bifidobacterium* (11.2%) were significantly increased while *Ruminococcaceae UCG014* (0.49%) and *Lianaceae RC9 intestinal group* (1.88%) significantly decreased in the WT‐CR group (*p* < 0.05) (Figure 10C).

## 4 DISCUSSION

CR is considered one of the most effective and simple ways to delay aging and extend lifespan.^27^ Although previous studies have shown that CR can attenuate AD progression, the complete mechanism remains unclear,^13^ especially that few reports pointed out the intact pathway regarding how CR affect the brain–gut axis. In the present study, first, we found that CR effectively improved cognitive function in AD mice. In the water maze experiment, learning ability and spatial memory were significantly improved in AD mice after they received CR for 6 weeks. Second, we found that CR has reduced the levels of soluble Aβ40 and Aβ42. Soluble Aβ is neurotoxic and is one of the major causes of cognitive impairment. The level of soluble Aβ positively correlates with the severity of cognitive impairment.^28^, ^29^ Aβ is a polypeptide fragment generated by the hydrolysis of amyloid precursor protein (APP), a process that requires the participation of β‐secretase and γ‐secretase.^30^, ^31^ If APP is cleaved by α‐secretase, it will not generate Aβ peptide. On the other hand, when APP is cleaved by β‐ and γ‐secretase, it will result in the amyloidogenic pathway. Soluble Aβ, generated by APP hydrolysis, comprises two main forms: Aβ40 and Aβ42. Compared to Aβ40, Aβ42 forms pathological fibrils more promptly and has stronger neurotoxicity. Moreover, Aβ42 is the main component of insoluble amyloid plaques.^32^, ^33^ The presence of insoluble amyloid plaques is one of the characteristic pathological changes in AD, and is closely associated with disease severity.^34^ Therefore, soluble Aβ content and the percentage of positively stained amyloid plaque area can be used as indicators of the severity of AD. Interestingly, in the present study, a significant decrease of soluble Aβ levels in the hippocampus was observed after a 6‐week CR regimen in AD mice. We believe that CR decreases Aβ levels in the following two ways. First, CR leads to decreased production of APP. Second, CR inhibits the activity of the APP hydrolase or decreases the amount of this hydrolase. Overall, the mechanism by which CR improves AD is decreasing Aβ production.

In the current experiment, we found in the levels of neuroinflammatory factors and ROS in the hippocampus of 36‐week‐old AD mice after CR. CR significantly reduced the levels of neuroinflammatory factors and ROS in hippocampus of AD mice. Neuroinflammatory cytokines and ROS are important indicators of the pathological process in AD. Neuroinflammation mediated by glial cells is a common pathological reaction in AD, and is closely related to Aβ neurotoxicity.^35^ A large number of glial cells accumulate near amyloid plaques in AD patients and model animals, and this accumulation is accompanied by increased expression of inflammatory factors, pro‐inflammatory factors, and increased ROS levels.^36^, ^37^ The abnormal increase and aggregation of Aβ in the brain of AD patients would in turn induce abnormal activation of glial cells, and increase the expression of pro‐inflammatory cytokines, IL‐1β and TNF‐α. At the same time, pro‐inflammatory factors could increase ROS levels activating downstream signaling pathways, further promote nerve inflammation, and eventually lead to neuronal loss and aggravate cognitive impairment. Inhibition of neuroinflammation in the brain can effectively reduce the incidence of AD and improve its prognosis.^38^, ^39^ Thus, we determined the degree of neuroinflammation by detecting the pro‐inflammatory factors IL‐1β and TNF‐α, as well as ROS, which are involved in the regulation of neuroinflammatory responses in AD.^40^ Our experiment showed that after CR for 6 weeks, the expression of IL‐1β, TNF‐α, and the levels of ROS in the hippocampus of 36‐week‐old AD mice was significantly lower than that in the control group. This strongly suggests that CR could specifically improve the neuroinflammation of brain tissue.

In the past decade, numerous studies have revealed that gut microbiota may have a close relationship with the occurrence and development of AD. However, the specific mechanism is still unclear. As mentioned before, both the neuroinflammation and systemic inflammation serve as the key hubs to accelerate the process of AD by promoting pathology and damaging neuron. At the same time, the gut microbiota is also crucial for the regulation of inflammation.^41^ We found that CR treatment resulted in significant diversity in composition and abundance of gut flora. The gut microbiota is directly or indirectly involved in the regulation of peripheral and central inflammation.^42^ There exists microflora–brain interaction pathway, and the microbiota–gut–brain axis has been a hot topic in recent years, which could influence the process of AD.^22^ CR can promote the transformation of gut microflora from pro‐inflammatory type to anti‐inflammatory type in patients with AD. Probiotics or dietary changes not only provide substrates to the gut microbiota that promote the production of anti‐inflammatory products but also alter the composition of gut microflora.^43^ However, most of the current studies are limited to the association between calorie restriction, gut microbiota, inflammation, and AD. The inflammatory pathway is one of the significant bridges between each other. The components of gut microbiota, such as LPS and amyloid protein, have been proved to exist in the brain tissues and peripheral organs of AD patients. They stimulate immune cells such as microglia and monocyte/macrophages to secrete proinflammatory cytokines and accelerate the process of AD. In addition, abnormal contents of the metabolites (short‐chain fatty acids, branched amino acids, bile acids, and neurotransmitters) of gut microbiota also exist in patients with AD.^44^ Of the currently available therapeutics, we propose that CR is a most promising avenue for improving cognitive health.

Previous studies have found that AD patients often have intestinal flora changes, but the specific reasons are unknown. In physiological conditions, gut microbiota exerts its effects on the central nervous system using several mechanisms, including the production of short‐chain fatty acids, modification of blood–brain barrier permeability, modulation of specific neurotransmitters, and vagus nerve stimulation. Recent studies have also suggested that intestinal flora is involved in the regulation of inflammation, including inflammation of the nervous system. Intestinal flora affects intestinal immune activation and oxidative stress by affecting the production and absorption of intestinal inflammatory factors, and then affects the inflammation and oxidative stress response of the central nervous system.^45^ The pathophysiological changes of AD are closely related to oxidative stress and inflammation, so we speculate that intestinal flora can affect the occurrence and process of AD by affecting inflammation and oxidative stress. Our study showed that intestinal flora in the AD‐CR group was significantly changed compared with that in the AD group. Our results demonstrated that the attenuation of experimental Alzheimer's disease following CR treatment in APP/PS1 mice may result from alterations in the gut microbiome. In addition, gut flora could be a new target for AD prevention and therapy. Therefore, we can speculate that CR is very likely to play the protective role on AD by changing intestinal flora and affecting the inflammatory state of the nervous system.

Lastly, we would like discuss the animal model we chose for this study. Interestingly, Halagappa, Schafer, and Wang et al. used different CR models of food restriction (20%–40%) in the AD mice models.^46^, ^47^, ^48^ The former found that the cognitive function of the AD model mice was effectively improved, and the latter two found that the pathological changes in the AD model mice were alleviated. However, none of these studies evaluated the safety of this CR approach. Therefore, in the current study, we chose a 30% CR model, which is considered comfortable and safe.^11^ We measured mice body weight in each group during the experiment, and found that the greatest weight loss occurred in the CR group from week 2 to week 4 and ended after week 4. In addition, no malnutrition or abnormal deaths were observed in the CR group throughout the experiment, suggesting that the CR approach was safe. The AD transgenic animal models can be established by overexpression of single or multiple mutant proteins, such as APP, PS1, PS2, Tau, and APOE.^49^, ^50^ APP/PS1 double transgenic mice, a classic model of AD with familial amyloid precursor protein and presenilin‐1 mutations, have been widely used in animal studies of AD.^51^, ^52^ We selected the APP/PS1 transgenic mice due to the close similarity of their pathological and clinical features with those of AD patients.

In clinical practice, the time when patients receive clinical treatment is usually when they are diagnosed with AD after they have developed cognitive impairment. Taking this into account, we established the intervention time point for animal experiments. Although increased extracellular soluble Aβ40 and Aβ42 expressions were detected in AD mice at the age of 12 weeks, cognitive decline was not obvious at this time point.^24^ AD mice showed significant impairment in learning, spatial memory, and executive ability at the age of 24–32 weeks.^53^, ^54^ Meanwhile, large deposits of Aβ plaques were detected in the corresponding brain areas. Interestingly, existing studies often chose to carry out food restriction at the very early stage when the AD model mice were at the age of 4–10 weeks, or started food restriction in the middle or late stage when the mice are 52–56 weeks old.^47^, ^55^, ^56^ Yet, we believed that the above intervention time points were either too early, or too late, which was not consistent with the intervention time point of AD at the clinical stage. We therefore chose to start the CR protocol at 30 weeks of age to match the early clinical stage of dementia in AD. This is also usually the first time when patients see a physician for cognitive decline..

In summary, CR could effectively inhibit the inflammatory responses, reduce the cognitive impairment in AD mice, and decrease Aβ expression. These protective effects may be closely related to alterations of the composition and diversities of gut flora.

## 5 CONCLUSION

Our study demonstrates that CR can reduce Aβ expression in the APP/ PS1 transgenic mouse model by suppressing inflammatory responses and oxidative stress, and CR results in the alterations in the gut microbiome leading to the attenuation of AD. Gut flora could be a new target for AD prevention and therapy.

## AUTHOR CONTRIBUTIONS

DZ and PL designed the experiments and revised the article. Junyu Chen and Cong Zou contributed equally to this work. JC and CZ performed the experiments and wrote the manuscript. HG guided experiments and the composition of the manuscript. XZ, LH, YC, and JX analyzed the data and searched the literature. All authors contributed to the revision of the manuscript and approved the submitted version.

## FUNDING INFORMATION

This work was supported by Natural Science Foundation of Guangdong Province (2023A1515011047). This work was supported by Natural Science Foundation of Guangdong Province (2019A1515011611). This work was supported by The Key Project of Basic Research of Shenzhen (JCYJ20200109113603854).

## CONFLICT OF INTEREST STATEMENT

The authors declare that the research was conducted in the absence of any commercial or financial relationships that could be construed as a potential conflict of interest.

## Data Availability

The data that support the findings of this study are available from the corresponding author upon reasonable request.
